# The anti-photoaging effect of C-phycocyanin on ultraviolet B-irradiated BALB/c-nu mouse skin

**DOI:** 10.3389/fbioe.2023.1229387

**Published:** 2023-08-22

**Authors:** Yali Zhou, Renao Bai, Yifeng Huang, Weina Li, Jiana Chen, Zhiyun Cheng, Xunxun Wu, Yong Diao

**Affiliations:** ^1^ School of Biomedical Science, Huaqiao University, Quanzhou, China; ^2^ Shantou Polytechnic, Shantou, China; ^3^ Haixia Hospital Affiliated to Huaqiao University, Quanzhou, China

**Keywords:** C-phycocyanin, skin photoaging, ultraviolet B, nanodispersions, proinflammatory cytokines

## Abstract

**Introduction:** C-phycocyanin (C-PC), a photosynthetic protein obtained from *Spirulina*, is regarded a highly promising commercially available biochemical. Numerous *in vitro* and *in vivo* studies have provided evidence of C-PC’s ability to mitigate the inflammatory response, alleviate oxidative stress, and facilitate wound healing. However, despite the existing knowledge regarding C-PC’s protective mechanism against cellular apoptosis induced by ultraviolet B (UVB) radiation, further *in vivo* experiments are needed to explore its anti-photoaging mechanism.

**Methods:** In this study, a UVB-induced skin photoaging model was established using BALB/c-nu mice, and the potential protective effects of topically administered c-PC were investigated by various molecular biology tools. In addition, a novel delivery system, C-PC nanodispersion, was developed to facilitate the transdermal delivery of C-PC.

**Results:** C- PC demonstrated significant anti-photoaging activities in the UVB-induced skin. The application of C-PC to the dorsal skin of the mice resulted in improved macroscopic characteristics, such as reduced sagging and coarse wrinkling, under UVB irradiation Histological analyses showed that C-PC treatment significantly decreased the symptoms of epidermal thickening, prevented dermal collagen fiber loosening, increased the hydroxyproline (Hyp) content and activities of antioxidant enzymes (such as superoxide dismutase, catalase, and glutathione peroxidase) in mouse skin, decreased malondialdehyde levels and expressions of inflammatory factors (interleukin-1α [IL-1α], IL-1β, IL-6, and tumor necrosis factor-α), reduced matrix metalloproteinase [MMP-3 and MMP-9] expressions, and inhibited the phosphorylation of c-Jun N-terminal kinase, extracellular signal-regulated kinase, and p38 proteins in the mitogen-activated protein kinase family.

**Discussion:** By analyzing the results of the study, a new drug delivery system, C-PC nano-dispersion, was proposed, and the anti-photoaging effect of C-PC and its mechanism were investigated.

## 1 Introduction

Endogenous and exogenous aging of the skin with time are inevitable. Endogenous aging is a natural physiological phenomenon that is mainly controlled by genetic factors and associated with the physical state of individuals. However, exogenous aging can be caused by smoking or exposures to ultraviolet (UV) radiation and harmful chemicals (Fonseca et al., 2023). UV irradiation can cause many skin-related problems including skin tissue damage, skin erythema, edema, dark skin, telangiectasis, rough skin, and aging (Lephart, 2016). One of the most significant environmental elements contributing to skin aging is UV (Lephart, 2016). Premature aging of the skin due to excessive UV exposure is defined as skin photoaging, and reducing the damage and other related problems caused by UV to the skin by administering medications or other treatments is known as anti-photoaging (Gonzales and Fuchs, 2017; Thulabandu et al., 2018). According to their wavelengths, UV radiations can be classified as UVA (320–400 nm), UVB (290–320 nm), and UVC (100–290 nm) (Rawlings and Matts, 2005). UVC cannot cross the ozone layer of the atmosphere and thus fails to reach the earth’s surface or cause harm to a person’s body. Therefore, sunlight reaching the earth comprises mainly UVA and UVB (Rawlings and Matts, 2005). UVB can induce more photoaging damage to the skin than UVA, and the biological effectiveness of UVA is only 0.1% of that of UVB (Longuet-Perret et al., 1998; Dai et al., 2011). To summarize, skin photoaging is primarily caused by UV radiation, and numerous studies have demonstrated that natural products with antioxidant and anti-inflammatory properties are useful in preventing skin photoaging (Pangestuti et al., 2021; Lee et al., 2022). Including these natural products in cosmetics and health can prevent skin photoaging, representing a new approach to the future prevention and treatment of skin photoaging. Therefore, the development of proteins with anti-photoaging properties has a broad prospect. C-Phycocyanin (C-PC), which was discovered in cyanobacteria, red algae, and cryptophytes, is a light-trapping and pigment-binding protein and participates in photosynthesis (Bai et al., 2023). C-PC consists of blue-colored phycocyanin (an open-chain linearly extended tetrapyrrole compound) bound to a carrier protein, with a thioether bond linking the carrier protein to phycocyanin (Romay et al., 2003). According to studies, C-PC is frequently used as a natural food coloring and has a broad spectrum of biological qualities, including anti-oxidant, anti-inflammatory, immunomodulatory, and anti-tumor properties. Romay et al. discovered that C-PC has antioxidant and anti-inflammatory properties as it was able to considerably reduce the levels of hydroxyl and oxygen radicals (Romay et al., 1998a). Paloma et al. later obtained comparable outcomes (Ge et al., 2006; Bermejo et al., 2008). It has been claimed that C-PC can sustain intracellular antioxidant enzyme activity by increasing intracellular levels of reduced glutathione to regulate iron-induced oxidative stress and exerting antioxidant effects (Bermejo-Bescós et al., 2008). Another study showed that C-PC could effectively reduce ear edema and decrease myeloperoxidase activity in male SD rats by scavenging oxygen free radicals and attenuating inflammatory responses without any toxic effects (Romay et al., 1998b). Chun-YanShen et al. revealed that C-PC can exert antiaging effects via boosting antioxidant enzyme activity as well as thymus and spleen indices (Shen et al., 2017). Its potent antioxidant and anti-inflammatory activities warrant further rigorous experiments on C-PC treatment for photoaging skin in the future. Delivering hydrophilic proteins via the skin is challenging because of the inherent barrier effect of the skin (Anselmo et al., 2019). In response to the skin barrier problem, previous studies have successfully enhanced the penetration of protein-based drugs into the skin by using solids-in-oil (S/O) nanodispersion systems (Tahara et al., 2008; Hardiningtyas et al., 2018; Hardiningtyas et al., 2019). In *in vitro* skin penetration studies, Safrina et al. developed a C-PC transdermal protein delivery system based on S/O nanodispersion technology and found that it promoted C-PC delivery through the cuticle layer of Yucatan micropig skin (Hardiningtyas et al., 2019). Although the role of C-PC in inducing HO-1 expression via activation of the PKC α/β II-Nrf-2/HO-1 pathway and prevention of primary skin cells' apoptosis when exposed to UVB has been reported, further *in vivo* studies have not been performed, and to determine its anti-photoaging efficacy and underlying mechanism of action, additional research is required (Kim et al., 2018). Meanwhile, the barrier effect of the skin layer making it is difficult for the transdermal administration of hydrophilic macromolecules such as peptides and proteins, and this situation is a challenge that needs to be overcome in the development of current drug delivery systems. In previous studies, the use of S/O nanodispersion technology could increase the penetration of protein drugs into the skin, where hydrophilic protein molecules could effectively penetrate the hydrophobic stratum corneum (SC) layer through an oil base (Tahara et al., 2012; Kitaoka et al., 2015; Hardiningtyas et al., 2019). In this method, surfactant–protein complexes in which the protein is encapsulated by hydrophobic surfactant molecules are first prepared and the modified protein can be highly distributed in the desired oil phase (Pachioni-Vasconcelos Jde et al., 2016). The S/O system is advantageous because it does not require any specialist tool or skin preparation that is often required for other transdermal drug delivery systems involving the use of microneedles, electroporation, and jet injection (Yang et al., 2022). According to these investigations, S/O nanodispersions offer a wide range of prospective uses in the medical device and cosmetics sectors.

**FIGURE 1:**
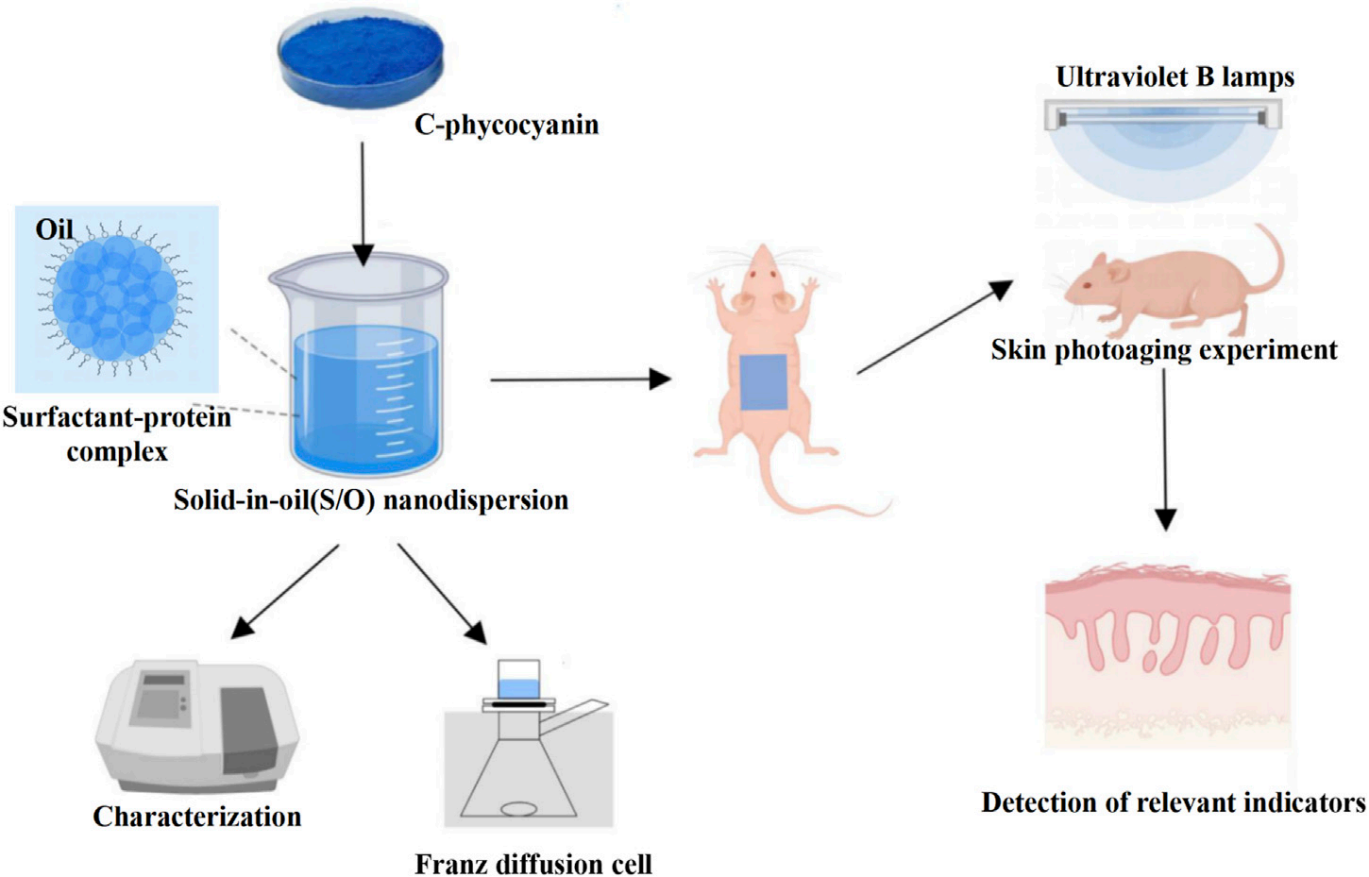
Schematic diagram of the anti-photoaging effects on UVB-exposed BALB/c-nu mouse skin treated with C-PC. C-PC nanodispersions were first prepared and characterized and then topically applied on the UVB-exposed photoaging model of BALB/c-nu mice; their anti-photoaging activity was evaluated by measuring their relevant physiological parameters such as antioxidant enzyme activities and inflammatory factor levels.

In this study, the dorsal skin of BALB/c-nu mice was topically treated with C-PC nanodispersions to examine its anti-photoaging efficacy. This study provides a certain reference value and experimental basis for the development of C-PC transdermal drug delivery system, and lays the foundation for the development of safe, efficient, and all-natural anti-skin photoaging agents. The flowchart shows the experimental process in detail (Figure 1).

## 2 Materials and methods

### 2.1 Chemicals and reagents

C-PC was purchased from Zhejiang Binmei Biotechnology Co., Ltd. (Taizhou, China). Sucrose ester (ER-290) was provided as a gift by Mitsubishi-Kagaku Food Co., Ltd. (Nanchang, China). Superoxide dismutase (SOD), glutathione peroxidase (GSH-Px), catalase (CAT), malondialdehyde (MDA), and hydroxyproline (Hyp) assay kits were obtained from Beijing Solarbio Science and Technology Co., Ltd. (Beijing, China). The BCA protein assay kit was procured from Beyotime Biotechnology Co., Ltd. (Shanghai, China). Antibodies specific for tumor necrosis factor-α (TNF-α), interleukin-1α (IL-1α), IL-1β, IL-6, matrix metalloproteinase-1 (MMP-1), MMP-3, and MMP-9 were provided by Wuhan Servicebio Technology Co., Ltd. (Wuhan, China). Jun N-terminal kinase (JNK), extracellular signal-regulated kinase (ERK), p38, phosphorylated JNK (p-JNK), p-ERK, and p-p38 and their specific antibodies were purchased from Proteintech Group, Inc. (Wuhan, China). Shanghai Aladdin Biochemical Technology Co., Ltd. (Shanghai, China) provided all additional reagents utilized in this investigation, all of which were of analytical grade.

### 2.2 Preparation of C-PC nanodispersions

C-PC nanodispersions were prepared using the S/O dispersion technique reported in previous studies (Tahara et al., 2012; Martins et al., 2013; Hardiningtyas et al., 2019). Briefly, 2 mL C-PC water solution (5%, wt%) was mixed with 4 mL ER-290 chloroform solution (125 mg/mL) and then sonicated on an ice bath for 5 min. Water in oil emulsions formed in the previous step was freeze-dried. The obtained freeze-dried samples were dissolved in isopropyl myristate to the final concentration of 2.5 mg/mL C-PC and stored at −20°C for further analyses and animal experiments.

### 2.3 Determination of the encapsulation efficiency and particle size of the C-PC nanodispersions

Lyophilized C-PC nanodispersions were suspended in 5 mL cold phosphate-buffered saline (PBS, pH 7.4). After a thorough shaking of the mixture, the supernatant was centrifuged at 18,000 g for 10 min at 4°C and removed for further examination. Using a UV-3802 (Unico, Wisconsin, United States) spectrophotometer, the free C-PC concentration in the supernatant was quantified and examined. The concentrations of C-PC (Bennett and Bogorad, 1973; Boussiba and Richmond, 1979; Luo et al., 2016) and the encapsulation efficiency (EE) were calculated as follows (Hardiningtyas et al., 2018):

$$C_{C-PC}\;\left(mg/mL\right)=\left({OD}_{620}-0.0474\left[{OD}_{650}\right]\right)\;/\;5.34$$

$$EE\;\left(\%\right)=\left(\;{\left[C_{C-PC}\right]}_{feed}\;*\;V-{\left[C_{C-PC}\right]}_{supernatant}\right)\;*\;V\;/\;{\left[C_{C-PC}\right]}_{feed}\times 100$$

The particle size of the nanodispersions was determined via dynamic light scattering (DLS) analysis using a nanoparticle size analyzer (Brookhaven Instruments Corporation, New York, United States). The prepared nanodispersions were diluted with isopropyl myristate (IPM) until they turned colorless, followed by the addition of an appropriate amount of the sample to a 1-mL quartz cuvette for particle size determination.

### 2.4 Morphological observations of the C-PC nanodispersions

The surface morphologies and particle sizes of the C-PC nanodispersions were characterized using the H-7650 transmission electron microscope (TEM) (Hitachi Limited, Tokyo, Japan) (Bermejo-Bescós et al., 2008; Okuma et al., 2009). The C-PC nanodispersion was diluted 20 times with IPM solvent and transferred to a carbon-coated copper grid. Then, the samples were stained with 2% of phosphotungstic acid. The excess stain was removed by using filter papers, and then, the samples were dried for a short while at room temperature. The prepared samples were subjected to TEM to obtain high-resolution TEM images at an accelerated voltage of 100 kV.

### 2.5 *In vitro* transdermal studies of the C-PC nanodispersions

BALB/c-nu mice (6–8 weeks; 18–22 g) were provided by Wu Laboratory Animal Trading Co. The National Institutes of Health Guide for the Care and Use of Laboratory Animals served as the standard for all experimental protocols, and the Huaqiao University Animal Care and Use Committee approved all animal experiments (grant number: A2022042). The mice were housed in SPF-compliant animal facilities at a temperature of 22°C with a 12-h light/dark cycle and free access to food and water. The BALB/c-nu mice were euthanized by cervical dislocation; the dorsal skin was excised, and the subcutaneous tissue as well as fat were removed carefully. Excised skin was cut into sections of appropriate sizes. *In vitro* skin permeability of the C-PC nanodispersions was assessed using the Franz Cell method (Hardiningtyas et al., 2019). The mouse skin was fixed with the epidermis on the top and the dermis on the bottom. A supply chamber was placed on top of the epidermis, keeping the dermis in direct touch with the receiving solution. The receiving cell’s volume was 10 mL, and its effective area was 0.8 cm^2^. PBS with a pH of 7.4 was used to fill the receiving cell. The magnetic stirrer is turned on and maintained rotating at 500 revolutions per minute while the receiving cell is positioned in a constant temperature water bath at 35°C. The supply chamber was filled with the aqueous C-PC solution or C-PC nanodispersions containing 2.5 mg/mL C-PC (300 µL), sealed with a sealing film, and then covered with aluminum foil. After 24 h, the apparatus was turned off, the mice’s skin was removed, the effective skin in contact with the drug was retained and the rest was excised, and the excess drug was rinsed off with saline and dried. The skin was dissolved with a cryo-embedding agent, optimal cutting temperature compound, and immediately put into liquid nitrogen to freeze. Using a cryosectioner set to an 18-μm thickness, the frozen skin samples were divided into sections, sealed, and analyzed under a confocal laser scanning microscope (CLSM) (Nikon, Tokyo, Japan). Using a fluorescence microscope and an excitation wavelength of 646 nm, the samples were seen.

### 2.6 *In vivo* animal experiments of the C-PC nanodispersions

This section established an experimental model of photoaging animals with reference to the method described by Lee et al. (Lee et al., 2014). BALB/c-nu mice are nude and hairless, which can eliminate the skin irritation caused by hair removal, and thus were chosen as the animal model in the present study. In total, 24 specific-pathogen-free female BALB/c-nu mice (weight 18–22 g and aged 6–8 weeks) were randomly assigned to one of the following four groups: control group (no UVB irradiation; negative control group); model group (UVB-irradiated group; no drug treatment; model group); solvent group (UVB-irradiated group; IPM solvent before irradiation; solvent group); and C-PC group (UVB irradiation, with the administration of C-PC nanodispersions before irradiation). After 1 week of domestication, mice in all groups except the Control and Model groups were administered 200 μL of the corresponding drug on the back and then irradiated with UVB after 30 min of action. Two UVB lamps with a peak irradiance of 313 nm arranged in parallel and placed 30 cm above the mice served as the UVB source. An UV energy meter (UV-Integrator150, Shenzhen Excellence Instruments Co., Ltd., Shenzhen, China) was used to measure the irradiance (1 mJ/cm^2^). The mice’s dorsal skin was exposed to radiation five times a week, with an intensity of 100 mJ/cm^2^ (minimum erythema dose) for the first week, 200 mJ/cm^2^ for the second week, 300 mJ/cm^2^ for the third week, and 400 mJ/cm^2^ for the fourth week; the intensity of 400 mJ/cm^2^ was maintained until the end of the eighth week. At the end of the eighth week, the mice were sacrificed by cervical vertebrae, and the skin was promptly severed from the irradiated dorsal midsection of the mice. Saline-moistened cotton was used to remove the adherent fat and hoof tissue under the skin. The removed skin was divided into two parts and placed in paraformaldehyde for fixation and then stored at −80°C for subsequent experiments.

### 2.7 Histological examinations

Skin tissues were preserved in 4% neutral formaldehyde for 24 h, then eluted with an ethanol gradient, xylene-clear, paraffin-embedded, and cut into sections of 4-μm thickness. Epidermal proliferation was assessed using hematoxylin and eosin (H&E) staining. Masson staining was used to quantify the deposited collagen. The stained sections were imaged under a light microscope (Nikon, Tokyo, Japan) and measured using Image-pro plus 6.0 software.

### 2.8 Measurements of protein content

Protein concentrations were determined by referring to previous studies (Moore et al., 2010). The manufacturer’s instructions were followed while using commercial kits (Beyotime Biotechnology Co., Ltd. Product code P0012, visible spectrophotometric method). Based on the sample quantities, 50 volumes of BCA reagent A were mixed with 1 volume of BCA reagent B (50:1) to create the correct volume of BCA working solution. This mixture was then well mixed. Standard protein solutions (Bovine Serum Albumin [BSA]) (20 μL for each well) with concentrations ranging from 0 to 0.5 mg/mL were added to the wells of a 96-well plate. The wells of the 96-well plate were also filled with sample solutions or diluted sample solutions (20 μL each), which were then mixed with 200 μL of the BCA working solution and the mixture was allowed to stand for 30 min at ambient temperature (25°C). After obtaining absorbance readings at 562 nm, the protein contents of the samples were evaluated using the standard curve.

### 2.9 Measurements of Hyp, MDA, SOD, and CAT activities and GSH-Px levels in skin tissues

After being homogenized in 0.9% saline (1/9, m/v), the dorsal skin tissues of the mice were centrifuged at 8,000 g for 10 min at 4°C. The levels of Hyp and MDA as well as the activity of SOD, CAT, and GSH-Px were measured in the supernatants by utilizing the appropriate test kits and doing all measurements in accordance with the guidelines provided by the manufacturer.

### 2.10 Immunohistochemical analysis

The skin tissues were fixed in 4% neutral formaldehyde for 24 h; dehydrated with ethanol and xylene; paraffin-embedded; and cut into 4 μm-thick sections. After antigen repair, 3% BSA was used to prevent nonspecific binding sites, and 3% hydrogen peroxide was used to halt the activity of endogenous peroxidase. The sections were then treated with primary antibodies (anti-TNF-α, anti-IL-1α, anti-IL-1β, and anti-IL-6) overnight at 4°C. After washing the tissue sections, secondary antibodies were applied and the sections were incubated for 50 min at ambient temperature (25°C). Then, the sections were washed with PBS, stained with 3,3′-diaminobenzidine, and counterstained with hematoxylin. The sections were then dehydrated and sealed and examined using a Nikon light microscope, followed by their quantification with the aid of the program Image-Pro Plus 6.0.

### 2.11 Immunofluorescence analysis

MMP expressions in the skin were quantified using the immunofluorescence assay. Skin tissues were preserved in 4% neutral formaldehyde for 24 h. The tissues were dehydrated with ethanol and xylene, paraffin-embedded, and cut into 4-μm thick sections. Nonspecific binding sites were blocked after the sections underwent antigen repair by soaking them in 3% BSA. The sections were then incubated overnight with primary MMP-1, MMP-3, and MMP-9 antibodies at 4°C. After being thoroughly cleaned, the tissue samples were incubated with secondary antibodies for 50 min at ambient temperature (25°C), protected from light. Following dehydration and sealing, the sections were examined using a Nikon fluorescent microscope and quantified using Image-pro plus 6.0 software.

### 2.12 Western blotting

The skin tissues were cut into sections, placed in a centrifuge tube, and stored at −80°C. A cell lysis solution (RIPA/protease inhibitor/phosphatase inhibitor, 99 µL/1 µL/1 µL) was prepared, which was added at a tissue/cell lysis solution ratio of 1 mg/10 µL. A hand-hold high-speed homogenizer was used to improve the efficiency of lysis with a speed of 8,000 rpm (Beijing, China). To create the whole protein sample solution, the supernatant obtained from the centrifugation at 12,000 g for 10 min at 4°C was employed (Zhi et al., 2020). Following the manufacturer’s instructions, the BCA kit was used to calculate the protein concentrations. One volume of the sample was mixed with four times the volume of 5X protein loading buffer, denatured at 95°C for 8 min, cooled, and stored at −20°C. Gels were prepared in advance (10% separation gel and 5% concentration gel). After loading the electrophoresis bath as required, the denatured samples were added for electrophoretic separation at 120 V and subsequently transferred onto polyvinylidene difluoride membranes. The membrane was first incubated for 1 h with 5% skimmed milk, then with a primary antibody and an enzyme-labeled secondary antibody. Enhanced chemiluminescence reagents were used to create the protein bands. The Tanon-4600 chemiluminescence imaging system (Tanon, Shanghai, China) was utilized to take pictures of the protein bands, which were then quantified using ImageJ software. Tubulin was chosen as the protein internal reference.

### 2.13 Statistical analysis

The data were statistically analyzed using IBM SPSS 22.0 (IBM, Chicago, NY, United States), and one-way ANOVA was utilized for comparison. The mean and standard error of the mean are used to express all data collected for this study. A *p*-value <0.05 is considered to represent statistical significance.

## 3 Results

### 3.1 C-PC nanodispersion particle size and encapsulation rate

EE is a crucial indicator for the quality control of liposomes and nanoparticle-based drug delivery systems. Another important element for the effective creation of nanodispersions is particle size (Tahara et al., 2012). Nanoscale formulations enable materials to penetrate the skin’s structure and offer a significant surface area for quick medication release (Rao et al., 2009). Therefore, in this study, the EE and particle sizes were evaluated (Table 1). The mean particle size of the C-PC nanodispersions was determined to be 258.0 nm using the argon laser DLS method (488 nm), whereas the EE of the C-PC nanodispersions was 92.39%. The nanodispersions prepared with the addition of surfactant have a relatively low surfactant concentration, a particle size range of 100–500 nm, low viscosity, and a high stability to be a typical S/O nanodispersion (Tahara et al., 2012).

**TABLE 1:** The encapsulation efficiency and particle sizes of the C-PC nanodispersions.

C-PC (wt%)	Protein to surfactant ratio (w/w)	Co-solvent	Preparation method	Particle size (nm)	Encapsulation efficiency of C-PC (%)
5	1:5	Chloroform	Ultrasonic probe	258.0 ± 48.0	92.39 ± 1.21

### 3.2 TEM observation of C-PC nanodispersions

**FIGURE 2:**
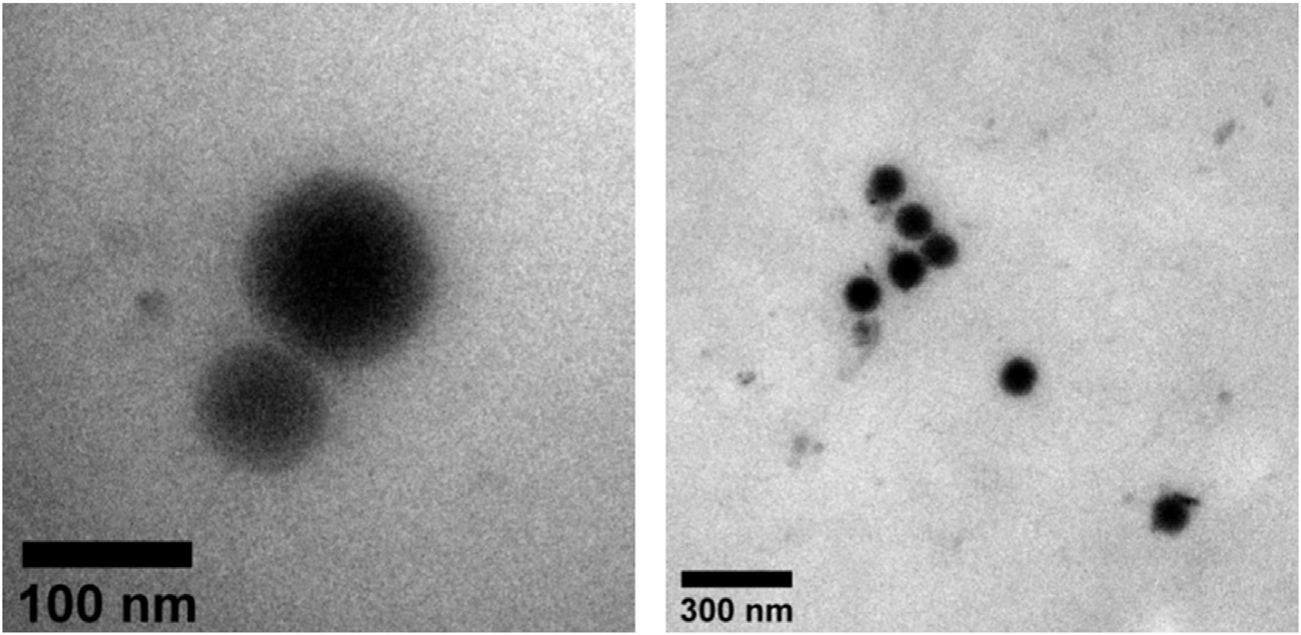
TEM views of C-PC nanodispersions.

The morphologies and surface characteristics of the C-PC nanodispersions were observed using TEM (Figure 2). The C-PC nanodispersions were spherical, and the particle sizes and diameters ranged from 100 to 300 nm. Furthermore, a collection of small substances could be observed in the particles, reflecting the C-PC encapsulated within the surfactant.

### 3.3 *In vitro* transdermal results of C-PC nanodispersions

**FIGURE 3:**
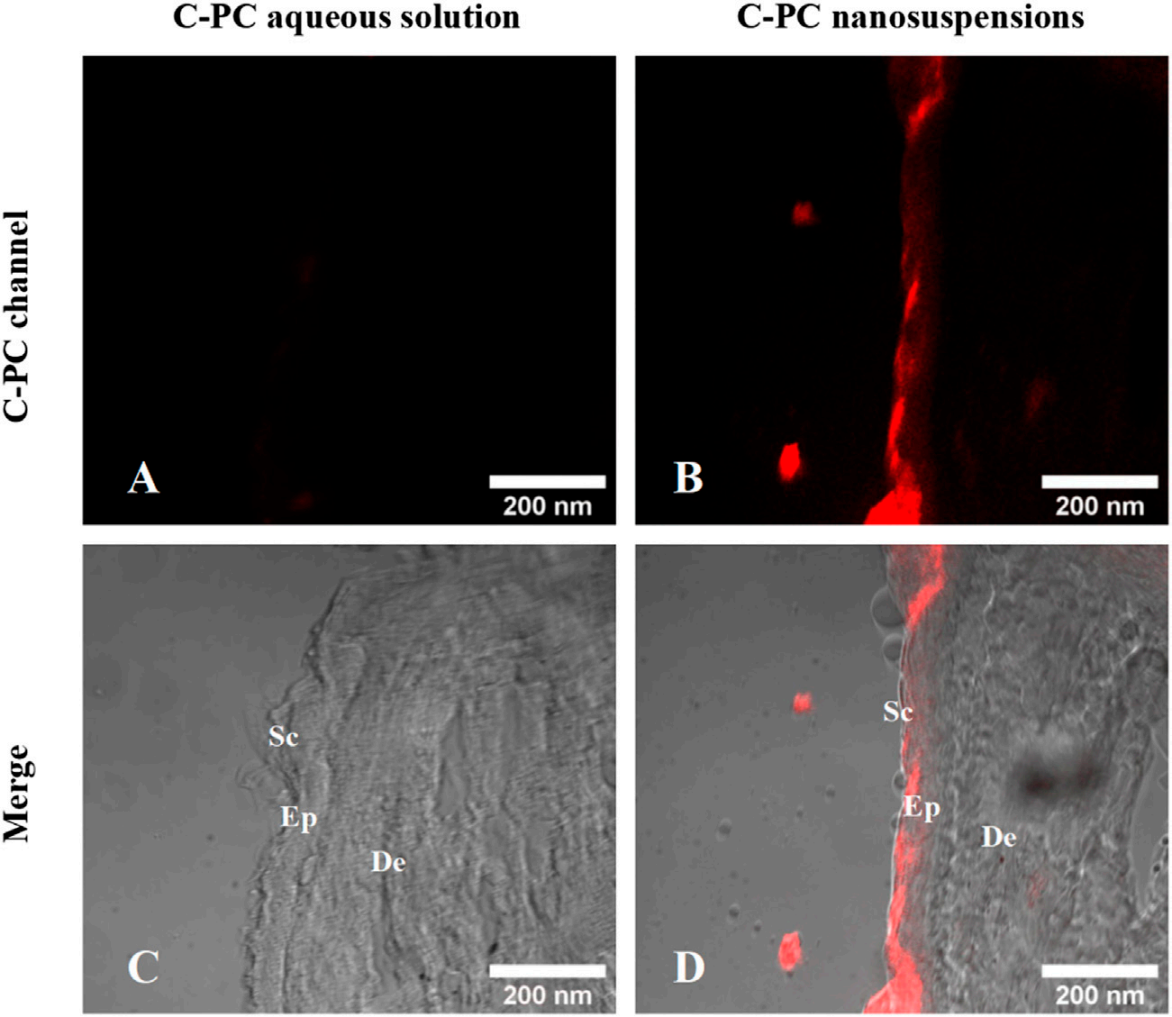
Confocal images of the frozen sections of BALB/c-nu mouse skin after a 24-h transdermal experiment. **(A)** C-PC fluorescence channel of C-PC aqueous solution. **(B)** C-PC fluorescence channel of C-PC nanodispersions. **(C)** Merging of C-PC fluorescence channels with the bright field of C-PC aqueous solution. **(D)** Merging of C-PC fluorescence channels with the bright field of C-PC nanodispersions. Sc, stratum corneum; Ep, epidermis; De, dermis.

Franz diffusion cells were established, and after 24 h of transdermal experiments, frozen sections of the experimentally treated mouse skin were obtained to observe the *in vitro* permeation behavior of C-PC aqueous solution and C-PC nanodispersions by using a CLSM (Figure 3). The intrinsic red fluorescence of C-PC at 639 nm indicated its presence in the skin. The fluorescence intensity of C-PC nanodispersions on the SC was much greater than that of the aqueous C-PC solution, indicating that C-PC encapsulated in nanodispersions and administered in an oil-based transport system could penetrate the main barrier including the SC layers of the skin.

### 3.4 Effect of C-PC nanodispersions on the macroscopic appearance of the skin and body weights of BALB/c-nu mice

**FIGURE 4:**
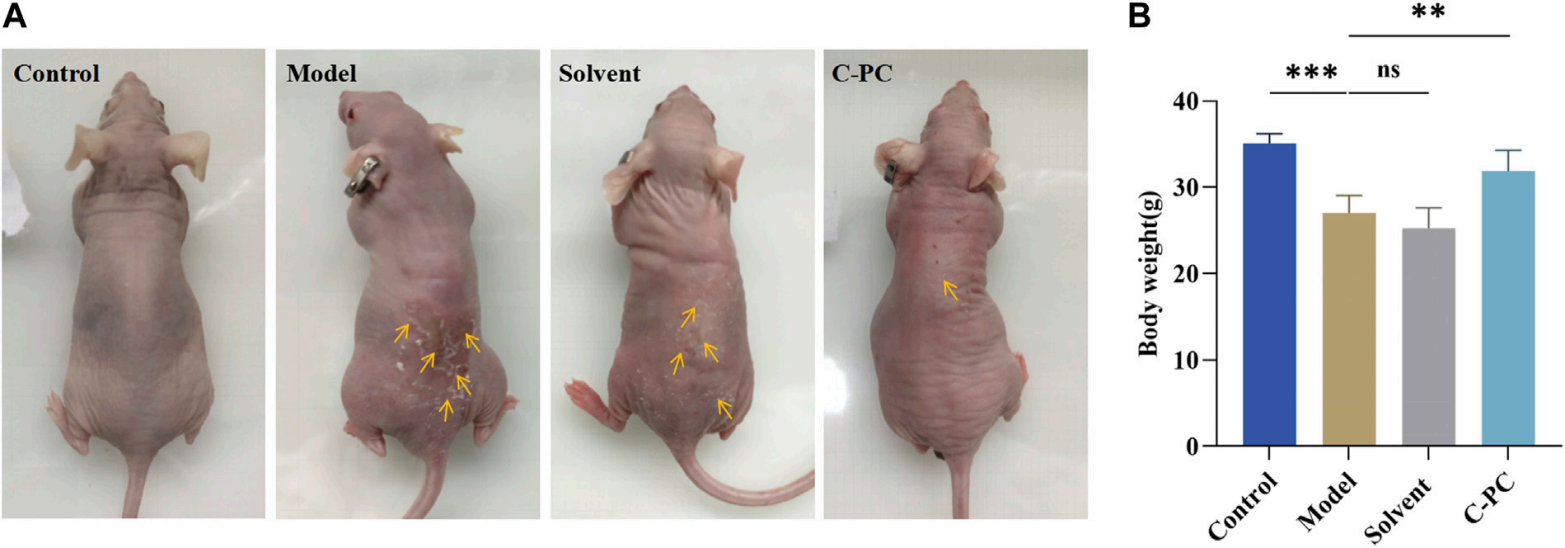
**(A)** Macroscopic skin performance of BALB/c-nu mice. **(B)** Body weight of mice at the end of 8-week photoaging experiment. Each value represents the mean ± SD (*n* = 6). ^∗^ *p* < 0.05, ^∗∗^ *p* < 0.01.

The control group mice exhibited smooth, undamaged, wrinkle-free, and elastic macroscopic skin appearance (Figure 4A). After 8 weeks of UVB exposure, the mice in the model and solvent groups showed dry, erythematous, coarse, and wrinkled skin with a leather-like appearance, slight surface damage, and distinct skin laxity. The skin damage, coarse wrinkles, and skin drooping in the C-PC group of mice were significantly reduced. This indicated that C-PC can reduce skin wrinkles, restore redness, reduce erythema, and alleviate photodamage. Compared to the mice in the control group, the body weight of the mice in the model group was noticeably lower. The mice in the C-PC group, however, had body weights that were noticeably different from those of the mice in the model group but similar to those of the mice in the control group (Figure 4B).

### 3.5 Effect of C-PC nanodispersions on skin thickness variation

**FIGURE 5:**
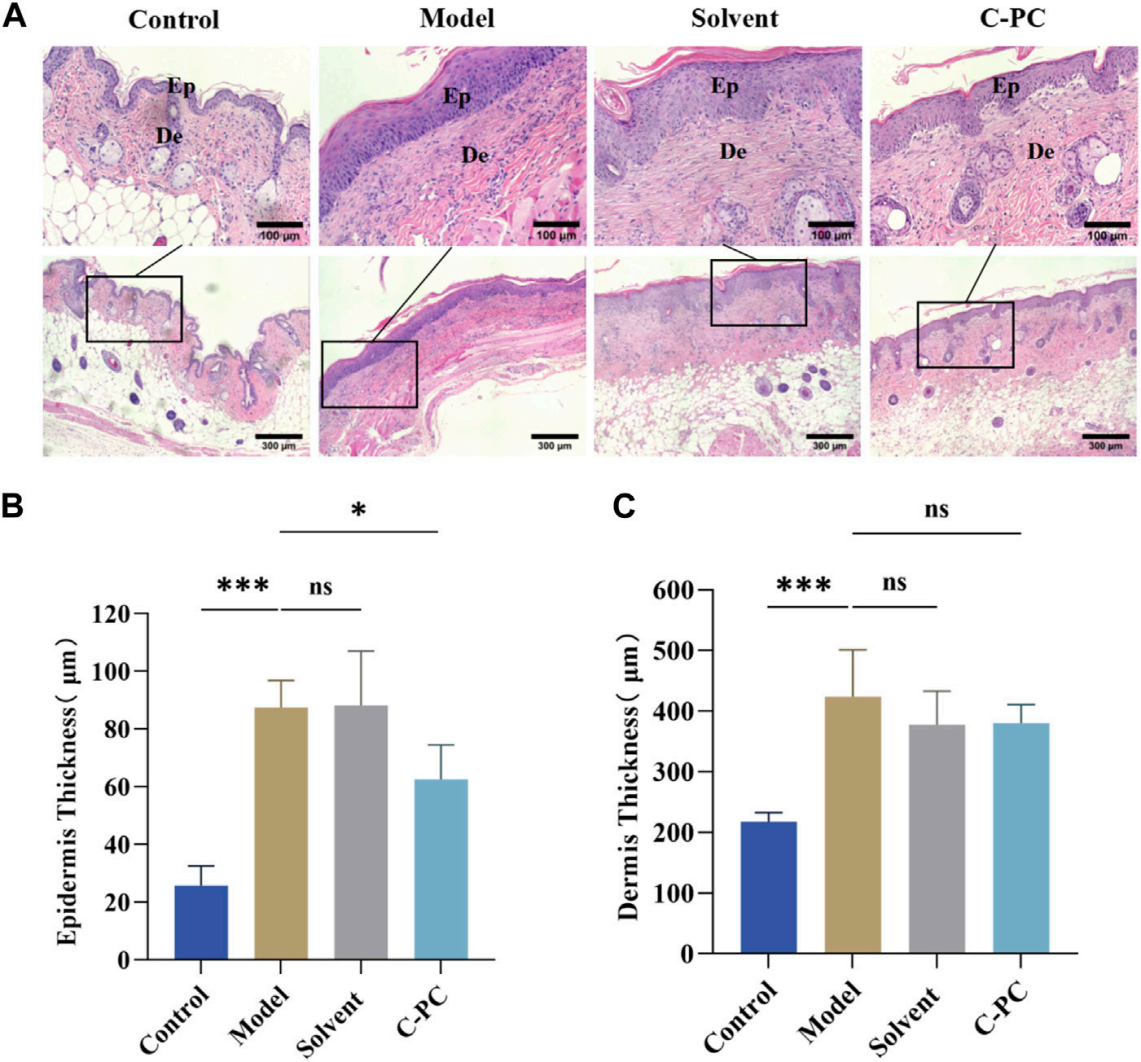
Hematoxylin and eosin (H&E) staining results of skin tissue from the BALB/c-nu mice (Scale bar: 100 μm). Ep, epidermis; De, the dermis. **(A)** H&E staining images. **(B)** Epidermal thickness analysis. **(C)** Analysis of dermal thickness. Each value represents the mean ± SD (*n* = 6). ns represents no significant difference, ^∗^ *p* < 0.05, ^∗∗^ *p* < 0.01, ^∗∗∗^ *p* < 0.001.

Continuous exposure of the skin to UVB induces skin inflammation, erythema, and epidermal thickening (Shah and Rawal Mahajan, 2013). H&E staining was performed on the skin tissue samples to examine the impact of C-PC on alterations in skin tissue after UVB exposure (Figure 5A). The control group had a thin epidermis with an intact and clear skin structure and a well-defined, wavy epidermal-dermal boundary. After UVB exposure, the mice in the model group had excessive cuticle and epidermal thickening, blurring of the dermal-epidermal boundary, and even inflammatory infiltration, indicating successful modeling of UVB-induced skin photoaging in the mice (Bosch et al., 2015). Mice in the solvent group had skin tissue in a comparable state to mice in the model group. In contrast to the model group, the C-PC group showed less structural skin damage and UVB-induced alterations. Additionally, the skin tissue state of the C-PC group matched that of the control group. Figure 5B displays the outcomes of a quantitative analysis of skin photoaging using epidermal thickness as a parameter (Lee et al., 2014). Contrasted with the control group, epidermal thickness was significantly higher in the model and solvent groups (*p* < 0.05). Epidermal thickness of the C-PC group was approximately 28.92% lower than that of the model group, with the difference being significant (*p* < 0.05), indicating that C-PC pretreatment had an inhibitory effect on UVB-induced epidermal thickening. The results of the dermal thickness analysis are presented in Figure 5C. Analysis of the results revealed that UVB irradiation caused a considerable thickening of the skin in mice compared to the control group, but there was no discernible thickening of the skin in the C-PC group compared to the model group.

### 3.6 Effect of C-PC nanodispersions on the morphology of dermal collagen fibers

**FIGURE 6:**
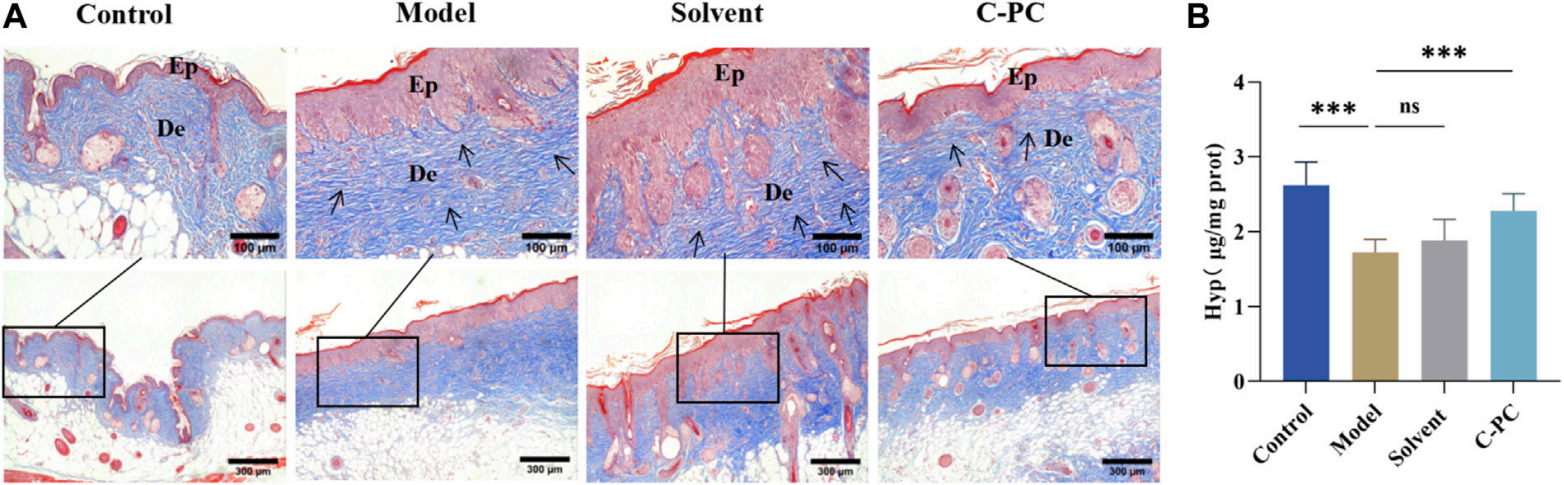
Skin collagen fiber morphology and Hyp content. **(A)** Masson staining results of BALB/c-nu mouse skin tissues (Scale bar: 100 μm). Ep, epidermis; De, dermis. **(B)** Hyp content of skin tissue in the BALB/c-nu mice. Each value represents the mean ± SD (n = 6). ns represents no significant difference, ^∗^ *p* < 0.05, ^∗∗^ *p* < 0.01, ^∗∗∗^ *p* < 0.001.

The primary constituents of the dermis of the skin are collagen fibers, which are vital for protecting and maintaining the skin’s structural integrity (Bosch et al., 2015). Masson trichrome staining of the skin tissue samples was performed to observe changes in dermal collagen fibers (Figure 6A), with dark blue staining for collagen fibers and red staining for cytoplasmic or muscle fibers. After UVB exposure, the skin collagen fibers of the control mice were tightly interwoven, thick, and evenly distributed, whereas those of the model and solvent groups were loose, irregular, distorted in arrangement, broken, or even abnormally piled up. The collagen fiber damage in the C-PC-treated group was less than that in the model group, the collagen fiber morphology was normalized, and the collagen fibers in the dermis were distributed more regularly and evenly, indicating that C-PC could protect the dermal collagen structure from UVB damage.

### 3.7 Effect of C-PC nanodispersions on Hyp content in mouse skin

The primary component of collagen fibers is Hyp, a special amino acid found in collagens (Kong et al., 2018). Hyp content is a measure of skin aging that directly reflects changes in the condition of collagen fibers in the dermis (Zhi et al., 2020). The effect of C-PC on Hyp contents in mouse skin is depicted in Figure 6B. Compared with the control group, the hyp contents in the model and solvent groups were significantly reduced (*p* < 0.05); however, the hyp contents in the C-PC group significantly increased compared with those in the model group. This result indicates that C-PC could significantly resist the decrease in Hyp content caused by UVB, inhibiting the degradation of collagen, promoting the formation of new collagen fibers in the skin, and increasing the skin repair function of damage.

### 3.8 Effect of C-PC nanodispersions on antioxidant enzyme activities and malondialdehyde levels in mouse skin

Antioxidant enzymes like SOD, CAT, and GSH-Px, which scavenge excess oxygen species (ROS) and guard against oxidative stress, have been demonstrated to be hindered by UVB irradiation of the skin. MDA is a by-product of lipid peroxidation (Ames et al., 1993). To determine the effect of C-PC on the antioxidant capacities of mouse skin, the activities of SOD, CAT, and GSH-Px and the MDA level were measured. Table 2 presents the results. UVB irradiation significantly reduced the activities of SOD, CAT, and GSH-Px by 59.05%, 49.25%, and 29.76%, respectively, in comparison to the control group. There were no appreciable differences in the activity of these three key antioxidant enzymes between the solvent group and the model group. Additionally, SOD, CAT, and GSH-Px activities of the C-PC group were 104.95%, 38.23%, and 20.46% higher than those of the model group, respectively. MDA levels in the model group were 157.81% higher than those in the control group, indicating a significant difference. The UVB-induced skin MDA level was dramatically lowered in the C-PC-treated group by 15.62% compared with the model group, indicating that C-PC can successfully fend off skin oxidative stress by boosting antioxidant enzyme activity and lowering lipid peroxidation.

**TABLE 2:** Effect of C-PC nanodispersions on skin antioxidant enzyme activity and malondialdehyde levels in BALB/c-nu mice.

Group	SOD (U/mg protein)	CAT (U/mg protein)	GSH-Px (U/mg prot)	MDA (nmol/mg protein)
Control	113.3 ± 19.5	13.4 ± 1.6	159.3 ± 12.0	6.4 ± 1.9
Model	46.4 ± 16.7^∗∗^	6.8 ± 1.2^∗∗^	111.9 ± 9.7^∗∗^	16.5 ± 1.8^∗∗^
Solvent	64.5 ± 20.1^∗∗^	6.2 ± 1.9^∗∗^	117.4 ± 7.0^∗∗^	13.7 ± 3.5^∗∗^
C-PC	95.1 ± 38.5^###^	9.4 ± 3.2^#^	134.8 ± 9.1^##^	7.4 ± 1.0^##^

Each value represents the mean ± SD (*n* = 6). ^∗^ *p* < 0.05, ^∗∗^ *p* < 0.01, compared with the control group; #*p* < 0.05, ##*p* < 0.01, ###*p* < 0.001, compared with the model group.

### 3.9 Effect of C-PC nanodispersions on the expressions of inflammatory factors in mouse skin

**FIGURE 7:**
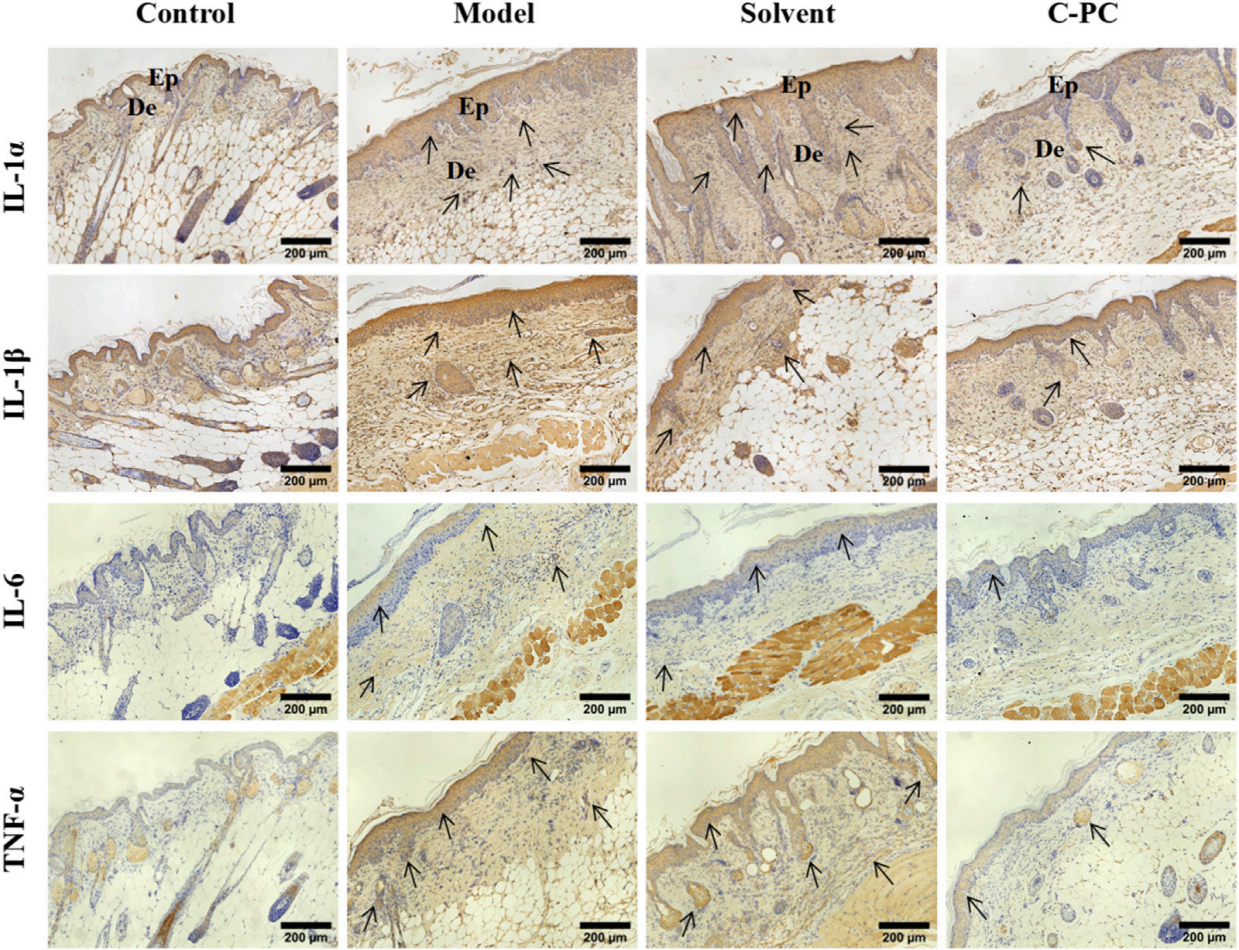
Immunohistochemical staining results for inflammatory factors (Scale bar: 200 μm). The positive signal for protein expression in immunohistochemical images of skin tissues is indicated in brownish-yellow color. Ep, epidermis; De, dermis.

**FIGURE 8:**
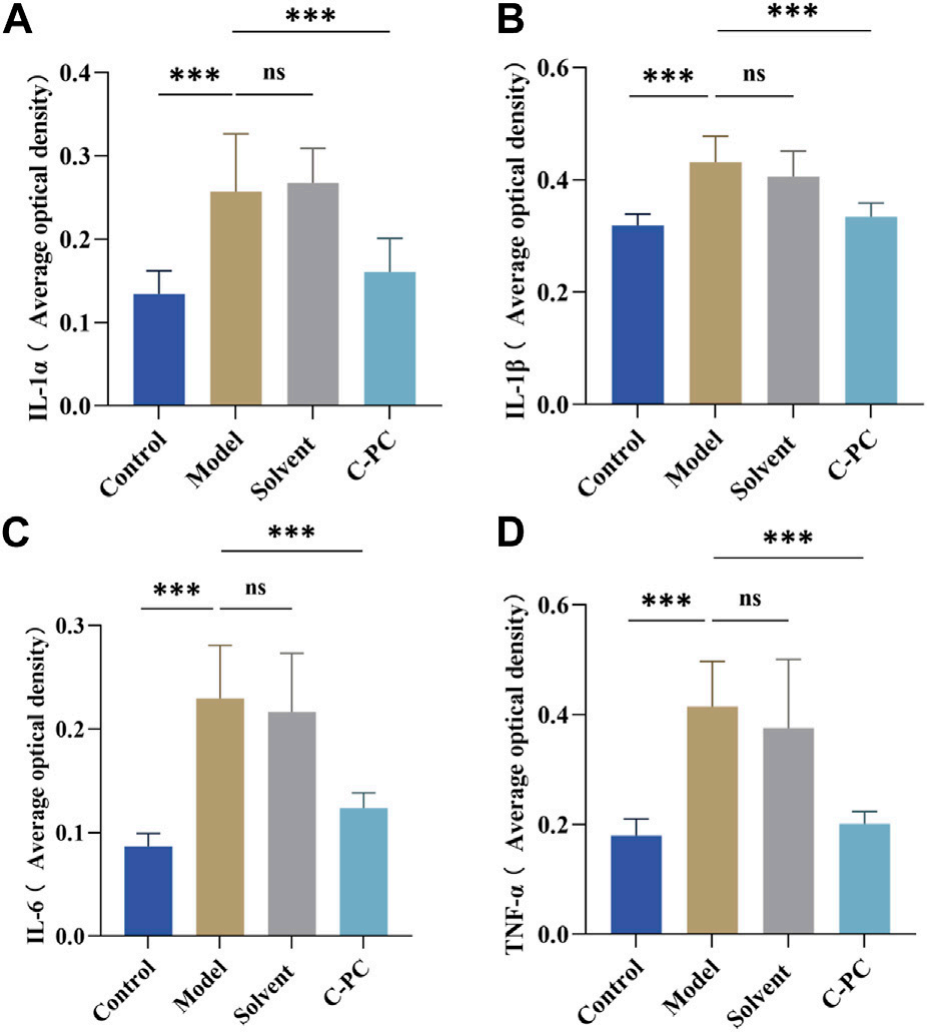
Results of the analysis of mean optical density values of inflammatory factors. **(A)** Quantitative analysis of IL-1α expression. **(B)** Quantitative analysis of IL-1β expression. **(C)** Quantitative analysis of IL-6 expression. **(D)** Quantitative analysis of TNF-α expression. The expression intensity was quantified using ImageJ 1.8.0 software. Each value represents the mean ± SD (*n* = 6). ns represents no significant difference, ^∗^ *p* < 0.05, ^∗∗^ *p* < 0.01, ^∗∗∗^ *p* < 0.001.

UVB radiation causes excessive ROS formation in cells, leading to oxidative stress, and encourages the release of additional proinflammatory hormones (Bosch et al., 2015). The impact of C-PC on UVB-induced expression and distribution of proinflammatory factors, including IL-1α, IL-1β, IL-6, and TNF-α, in mouse skin was assessed; the results are displayed in Figure 7. The results of the mean optical density value analysis are shown in Figure 8. After UVB irradiation, mice in the model and solvent groups significantly outperformed those in the control group in terms of the expression of positive signals in their skin tissues. Additionally, the mean optical density values varied significantly (*p* < 0.05), indicating that the expression of IL-1α, IL-1β, IL-6, and TNF-α was significantly upregulated. These results further indicated that C-PC can effectively reduce the levels of skin inflammatory factors, attenuate the inflammatory response, and inhibit anti-photoaging of the UV-exposed skin. The degree of staining and mean optical density values of IL-1α, IL-1β, IL-6, and TNF-α in the skin tissues of mice in the C-PC group were significantly lower than those in the model group (*p* < 0.05).

### 3.10 Effect of C-PC nanodispersions on MMP expression

**FIGURE 9:**
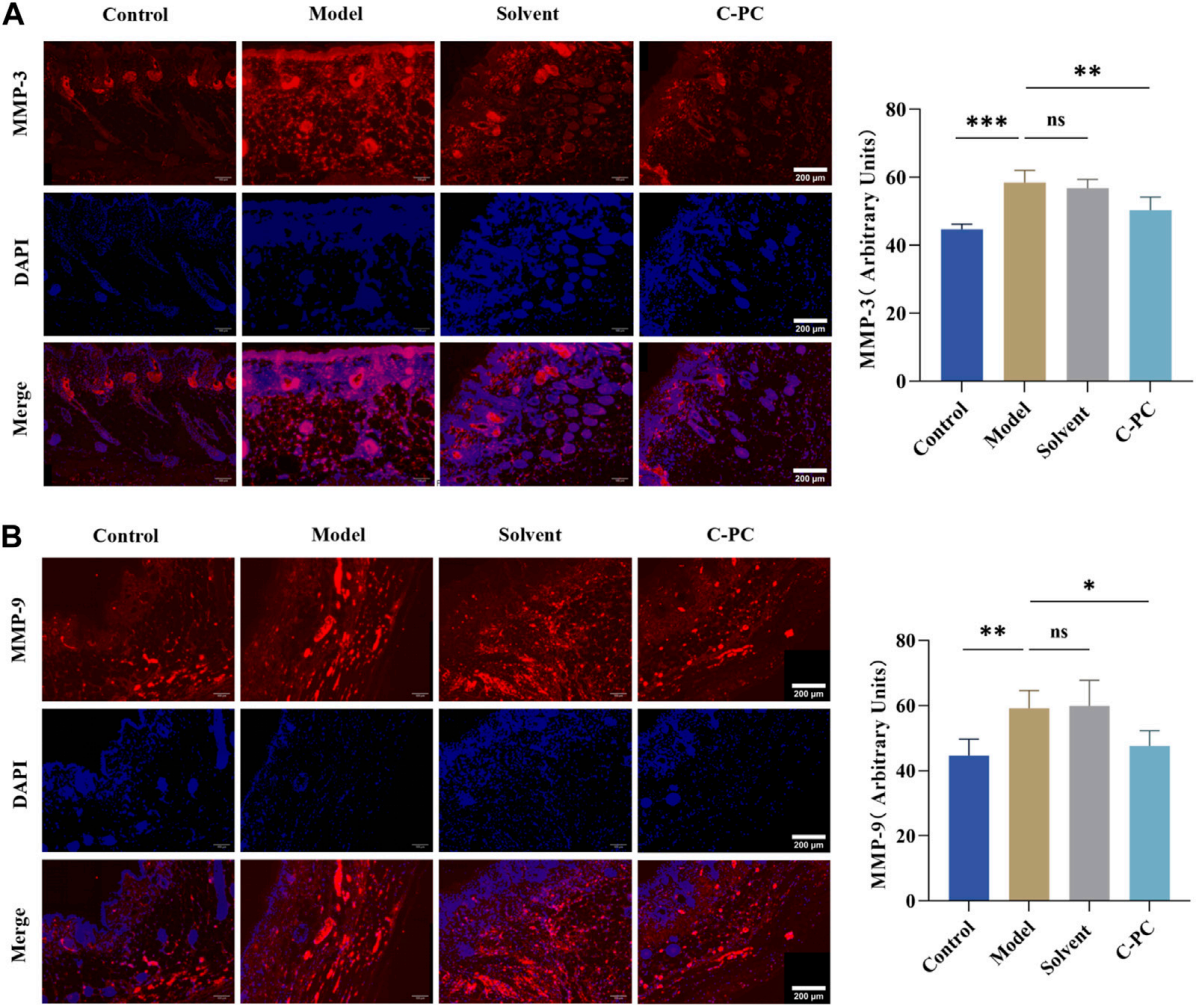
Immunofluorescence sections of MMPs (Scale bar: 200 μm) and quantitative analysis results. **(A)** Immunofluorescence and section quantitative analyses of MMP-3. **(B)** Immunofluorescence and section quantitative analyses of MMP-9. Each value represents the mean ± SD (*n* = 6). ns represents no significant difference, ^∗^ *p* < 0.05, ^∗∗^ *p* < 0.01, ^∗∗∗^ *p* < 0.001.

MMPs are mainly expressed in the dermis of the skin, especially in the matrix surrounding collagen fibers (Kim et al., 2012). The results of immunofluorescence expression and mean fluorescence intensity analysis of matrix metalloproteinases (MMPs) in each group are shown in Figure 9, with red fluorescence indicating MMPs and blue fluorescence indicating DAPI-stained nuclei. Comparing all other groups to the control group, the expression of MMPs increased to varying degrees, and the model and solvent groups exhibited significantly (*p* < 0.05) increased mean fluorescence intensity of MMP-1 (Supplementary Figure S1); MMP-3, and MMP-9. MMP-1 expression in the C-PC group was similar to that in the model group, but MMP-3 and MMP-9 expressions were low (*p* < 0.05). MMP-1 is a key player in the photoaging process because it starts the cleavage of fibrillar collagen’s core triple helix structure. In terms of time sequence, new cleavage of MMP-1 was followed by that of MMP-3 and MMP-9, while C-PC administration blocked the cleavage link guided by MMP-3 and MMP-9. This indicated that C-PC nanodispersions effectively inhibited the secretion of MMPs in the skin and protected the degradation of the matrix.

### 3.11 Effect of C-PC nanodispersions on the MAPK signaling pathway in mouse skin

**FIGURE 10:**
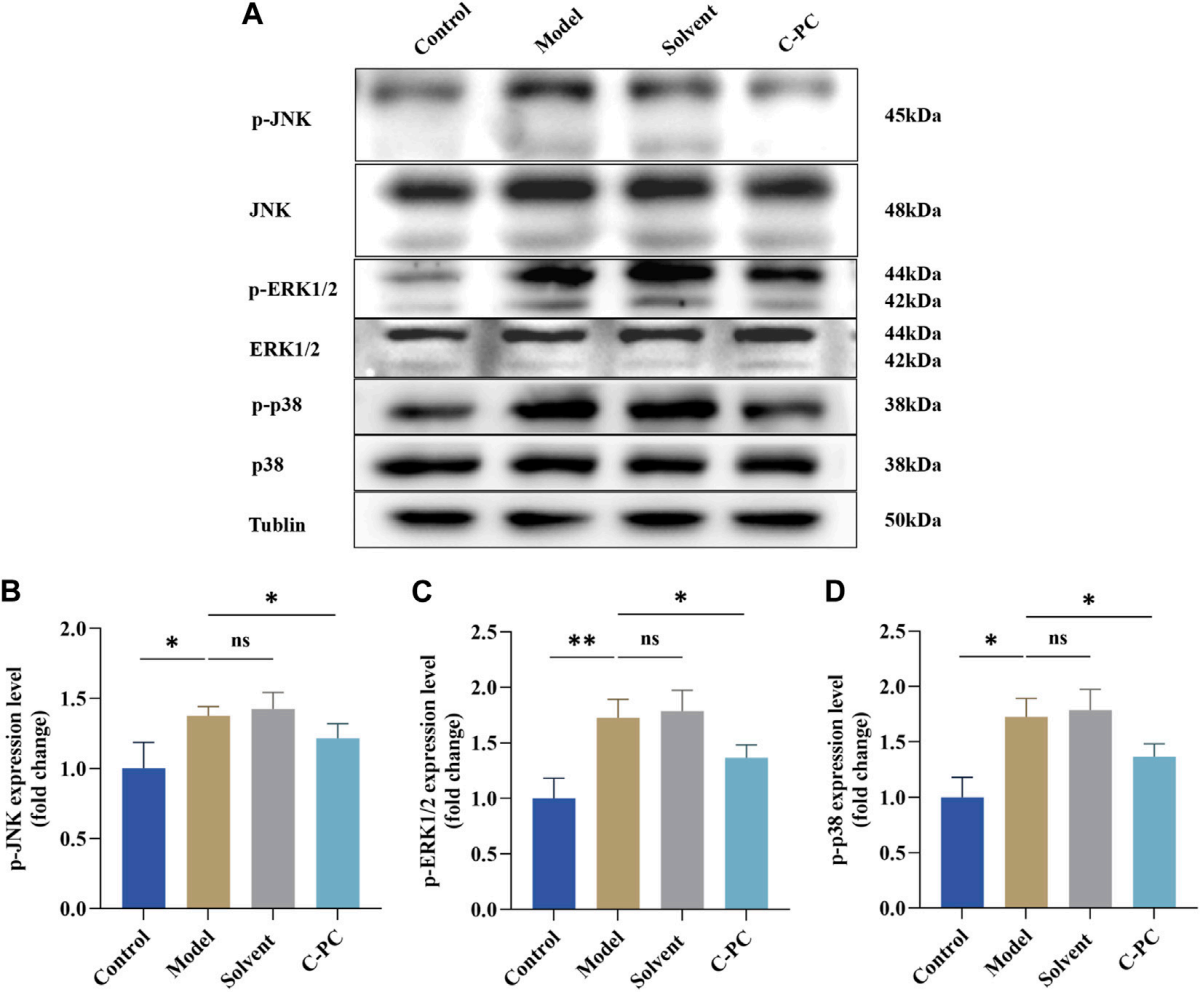
Effect of C-PC nanodispersions on the phosphorylation levels of MAPK family proteins in the dorsal skin of BALB/c-nu mice. **(A)** Phosphorylation expression of JNK, ERK1/2, and p38 protein. **(B)** Results of JNK phosphorylation expression analysis. **(C)** Results of ERK1/2 phosphorylation expression analysis. **(D)** Results of p38 phosphorylation expression analysis. The expression intensity was quantified using ImageJ 1.8.0 software. Each value represents the mean ± SD (*n* = 6). ns represents no significant difference, ^∗^ *p* < 0.05, ^∗∗^ *p* < 0.01, ^∗∗∗^ *p* < 0.001.

Western blotting was used to investigate how C-PC nanodispersions affected the mitogen-activated protein kinase (MAPK) signaling pathway in the skin of photo-aged mice. Figure 10 demonstrates that the UVB-induced phosphorylation levels of JNK, ERK, and p38 were significantly higher in the model and solvent groups than those in the control group by 1.36-, 1.72-, and 1.38-fold, respectively. These findings suggested that UVB irradiation increases the phosphorylation levels of JNK, ERK, and p38 proteins. Nevertheless, in contrast to the model group, UVB-induced p-ERK, p-JNK and p-p38 protein levels were significantly inhibited in the C-PC group (*p* < 0.05). These results suggested that UVB irradiation stimulates the phosphorylation of MAPK family genes, whereas C-PC inhibited UVB-induced JNK, ERK, and p38 activation, which may attenuate skin photoaging.

## 4 Discussion

In this study, proteins were initially encapsulated in hydrophobic surfactants to create surfactant–protein complexes. In the oil phase, the modified proteins were widely disseminated. Hydrophilic protein molecules can effectively traverse the hydrophobic SC layer in the oil base through the S/O nanodispersion mechanism. In addition, due to the small size and stability of S/O nano-dispersions, concentrations of proteins that can be dispersed in the oil base are higher than those of emulsifier-based vaccine carriers (Tahara et al., 2012). The skin tissue does not need any specialized tools or surface preparation when utilizing the S/O system, but they are frequently required for other transdermal drug delivery systems (such as those requiring microneedles, electroporation, and jet injections) (Yang et al., 2022). These studies suggest that the S/O nanodispersion system has strong potential applications in the pharmaceutical and cosmetic fields. In this study, C-PC nanodispersions were prepared successfully, and their physicochemical properties and transdermal effects were investigated. The results indicated that the prepared C-PC nanodispersions can penetrate the SC layer of the skin by using the oil-based transport system of the S/O nanodispersions.

Prolonged UVB radiation exposure results in skin photoaging and contributes to the dry, wrinkled, and saggy appearance of the skin (Yaar and Gilchrest, 1998). Collagen is an important constituent of the extracellular matrix (ECM). UV radiation exposure reduces the expression of the transforming growth factor, which has an impact on the route for collagen synthesis and inhibits collagen expression (Magné et al., 2006). At the same time, UV induces the expression of AP-1, which ultimately leads to a reduction in collagen synthesis by repressing the encoding and transcription of collagen genes (Li et al., 2018). Furthermore, some inflammatory factors, including ILs and TNFs, react with some key proteins in the MAPK pathways, leading to the overexpression of MMPs, decrease in the collagen and elastin contents, and degradation of structural proteins in the dermis that maintain skin elasticity, eventually causing ECM damage, loss of skin elasticity, dryness, lack of shine, and accelerating skin photoaging (Kang et al., 2005; Pittayapruek et al., 2016). Because of the improved standard of living and awareness of antiaging among people, the field of aesthetic science and technology research is booming, and increasing attention has been paid to skin photoaging prevention and treatment. Hyp is a crucial gauge of the severity of skin aging. It is an amino acid specific to skin collagen that promotes collagen regeneration and metabolic skin damage repair (Pittayapruek et al., 2016). MMP overexpression leads to degradation of the ECM, including collagen fibers, which is the main cause of wrinkles and sagging of the skin. Meanwhile, UVB irradiation leads to irregular thickening of the skin epidermis, which is a direct cause of wrinkling and roughness. In this study, BALB/c-nu mice exhibited typical photoaging features such as dry, rough, erythematous, coarse wrinkled and sagging appearance to the naked eye after 8 weeks of UVB irradiation. On the other hand, histological examination of the mice’s dorsal skin showed that the epidermis had greatly thickened, and that the collagen fibers were sparse, disorganized, and broken, indicating that UVB exposure of mouse skin successfully induced photoaging. In contrast, C-PC treatment effectively reduced UVB-induced skin photoaging, inhibited epidermal thickening, maintained collagen fiber morphology and arrangement, increased Hyp content, reduced skin relaxation and wrinkling, and normalized mouse skin. Furthermore, it significantly inhibited the expression of the proteins MMP-1, MMP-3, and MMP-9 in skin cells.

The reactive ROS level in the body is low and in dynamic equilibrium under normal circumstance (Yao et al., 2022). However, exposure to UV radiation can cause the body to produce abundant ROS, which cannot be removed by mitochondria in a timely manner. UVB radiation weakens the body’s antioxidant defense system, causing oxidative stress and a decline in antioxidant enzyme activity that hastens the aging process (Wlaschek et al., 2001). In addition, inflammatory factors are maintained at low levels in normal cells, and ROS can stimulate skin cells to release a large amount of inflammatory factors and trigger an inflammatory response (Fisher et al., 1996).

Various antioxidant enzymes are essential components of the body’s antioxidant defense system, and they help maintain a dynamic balance of free radical levels while repairing oxidative damages in the body (Bosch et al., 2015). Studies have reported that UVB irradiation resulted in excessive ROS production, severely disrupting the skin’s antioxidant defense system and resulting in oxidative stress, eventually leading to photoaging (Magné et al., 2006). MDA, a well-known indicator of oxidative stress, is a breakdown product of UVB-induced lipid peroxidation; its level was reported to remarkably increase in the skin of UVB-exposed mice (Bosch et al., 2015). In the current investigation, C-PC considerably ameliorated UVB-induced decline in the activity of antioxidant enzymes including SOD, CAT, and GSH-Px and an increase in MDA levels. Furthermore, C-PC possibly protected the skin’s antioxidant system against photoaging.

JNK, ERK, and p38 are members of the MAPK family, which is a significant regulator of the cell surface-to-nucleus signaling pathways and is essential for cell proliferation, differentiation, and death as well as for controlling the release of inflammatory cytokines (Yao et al., 2022). ERK is largely initiated by growth factor receptor activation, whereas JNK and p38 are typically induced by cytokine receptors and a variety of environmental stressors, such as UV radiation (Kang et al., 2005; Yao et al., 2022). During photoaging, UVB-induced oxidative stress mediates the phosphorylation levels of the MAPK pathway proteins (Chen et al., 2020). However, no studies have demonstrated that C-PC inhibits UVB-induced phosphorylation of the MAPK signaling pathway and exerts an anti-photoaging effect. In the current investigation, C-PC dramatically reduced the UVB-induced phosphorylation of MAPK proteins, including JNK, ERK, and p38, in mouse skin. The analysis of these results showed that C-PC, a key substance in the prevention and therapy of photoaging, might lessen UVB-induced oxidative stress by reducing UVB-induced activation of the MAPK signaling pathway. According to studies, continuous UVB exposure causes skin inflammation, which is linked to skin photoaging. Activation of the MAPK signaling pathway also increases the release of inflammatory proteins such as IL-1α, IL-1β, IL-6, and TNF-α. The intensity of inflammation is thought to be regulated by IL-1α, IL-1β, IL-6, and TNF-α (Chen et al., 2020). In the current research, C-PC treatment effectively inhibited UVB-induced overexpression of TNF-α, IL-1α, IL-1β, and IL-6 in mouse skin and suppressed the activation of the MAPK signaling pathway. In conclusion, C-PC inhibits UVB-induced pro-inflammatory cytokine production and prevents the activation of the MAPK signaling pathway, which can reduce skin inflammatory reactions. Moreover, MAPK signaling pathway activation can promote the expression of MMP-1, MMP-3, and MMP-9, which are crucial for breaking the collagen structure and damaging the ECM (Yao et al., 2022). By preventing the phosphorylation of MAPK proteins in the current investigation, C-PC therapy decreased the UVB-induced production of MMP-1, MMP-3, and MMP-9.

In summary, using the BALB/c-nu mouse photoaging model, the present study revealed that C-PC nanodispersions can reduce UVB-induced coarse wrinkling and sagging of the skin, inhibit UVB-induced epidermal thickening, increase Hyp content in mouse skin, increase the activities of antioxidant enzymes (such as SOD, CAT, and GSH-Px) in skin tissues, decrease MDA levels, inhibit the expression of inflammatory mediators (such as IL-1α, IL-1β, IL-6, and TNF-α), decrease the expression of MMP-1, MMP-3, and MMP-9, and inhibit the phosphorylation of JNK, ERK, and p38 proteins in the MAPK family signaling pathway. These findings demonstrate that C-PC may be an excellent candidate as a natural, safe anti-skin photoaging agent.

## Data Availability

The original contributions presented in the study are included in the article/Supplementary Material, further inquiries can be directed to the corresponding author.
