# Supplementary material for: The anti-photoaging effect of C-phycocyanin on ultraviolet B-irradiated BALB/c-nu mouse skin
Source: Front Bioeng Biotechnol. 2023 Aug 22;11:1229387. doi: 10.3389/fbioe.2023.1229387 (PMC10478087; doi:10.3389/fbioe.2023.1229387)
Supplement: Supplementary file 1 [file DataSheet1.DOCX]

Supplementary Material

The anti-photoaging effect of C-phycocyanin on ultraviolet B-irradiated BALB/c-nu mouse skin

**Yali Zhou ^1#^ , Renao Bai ^1#^ , Yifeng Huang ^2^ , Weina Li ^2^ , Jiana Chen ^2^ , Zhiyun Cheng ^1^ , Xunxun Wu ^1^ and Yong Diao ^1^***

*** Correspondence:**  diaoyong@hqu.edu.cn (Y.D.); Tel.: +86-595-2269-2516 (Y.D.)
^#^  These authors contributed equally to this work.

# Supplementary Data

## Supplementary Figures

**
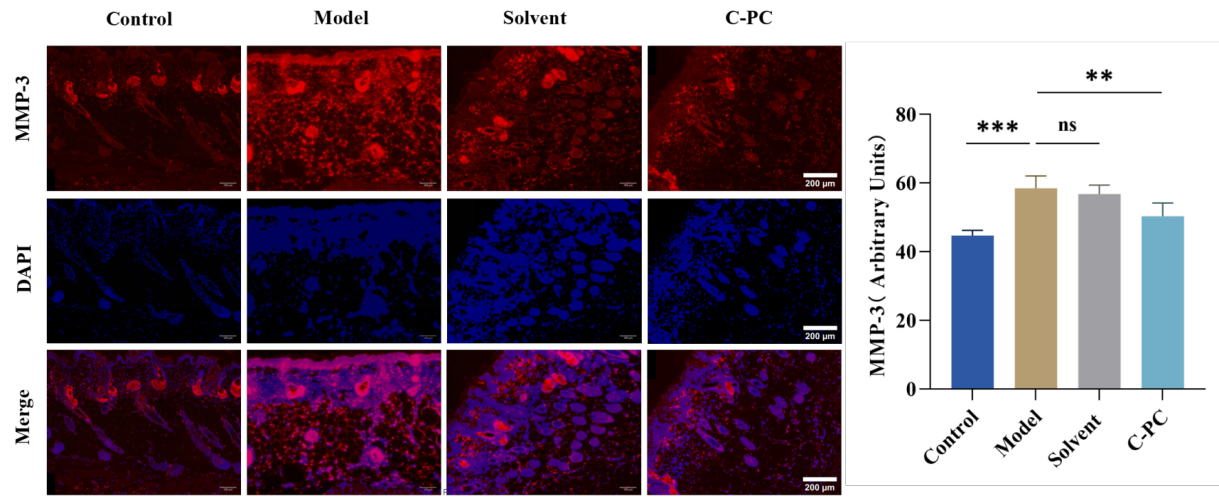
**

**Supplementary Figure 1.** Immunofluorescence and section quantitative analysis of MMP-1.

## WB raw data


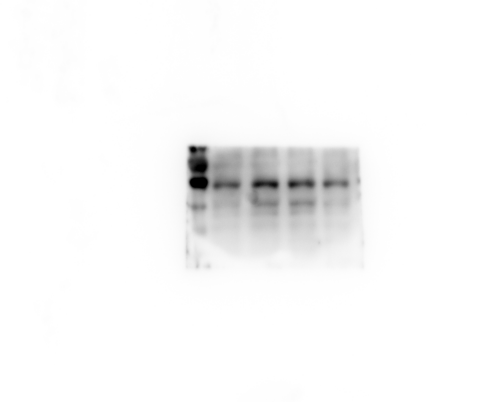


**Supplementary Figure 2.** WB raw data of p-JNK


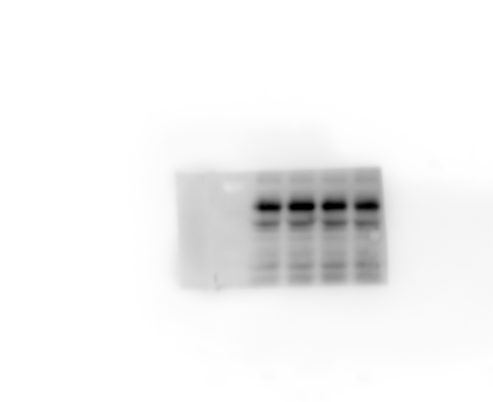


**Supplementary Figure 2.** WB raw data of JNK


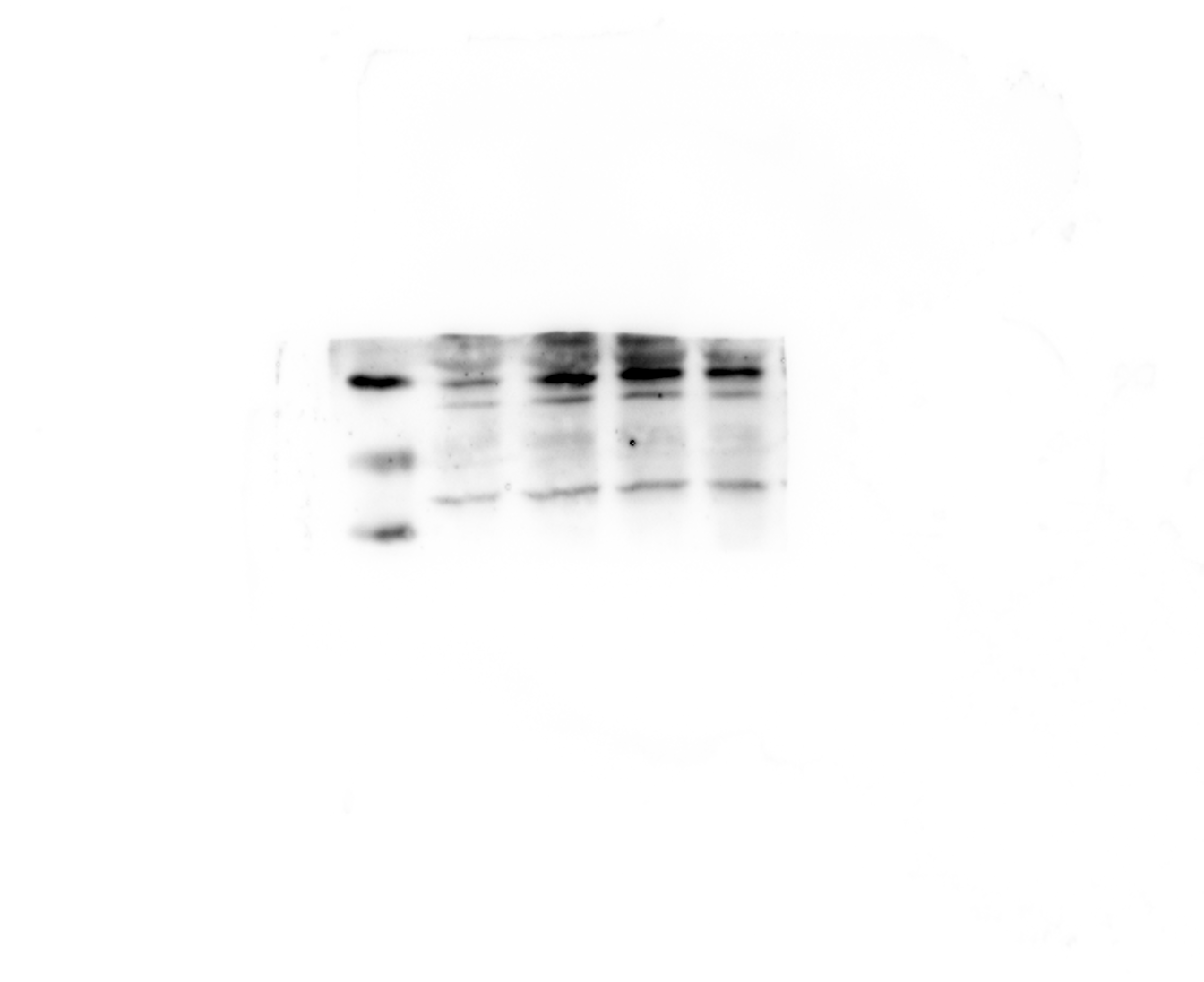


**Supplementary Figure 3.** WB raw data of p-ERK1/2


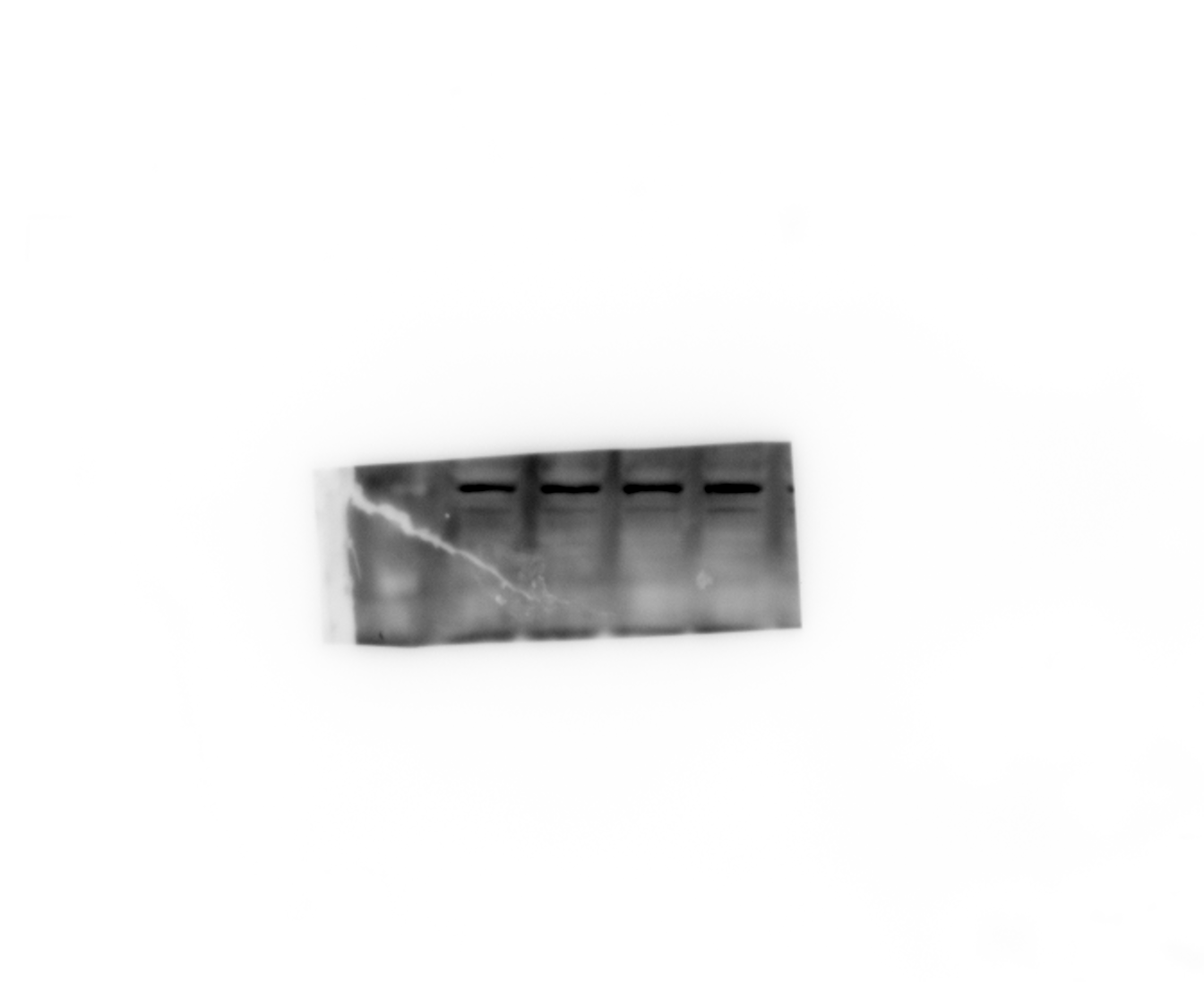


**Supplementary Figure 4.** WB raw data of ERK1/2


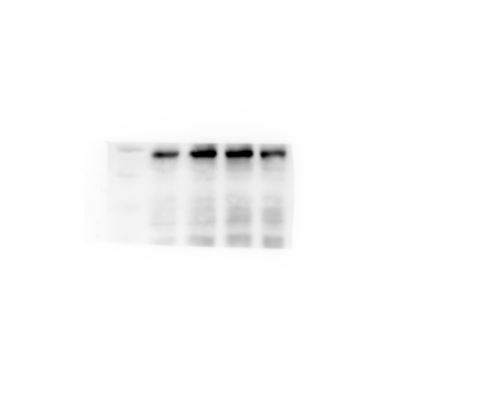


**Supplementary Figure 5.** WB raw data of p-p38


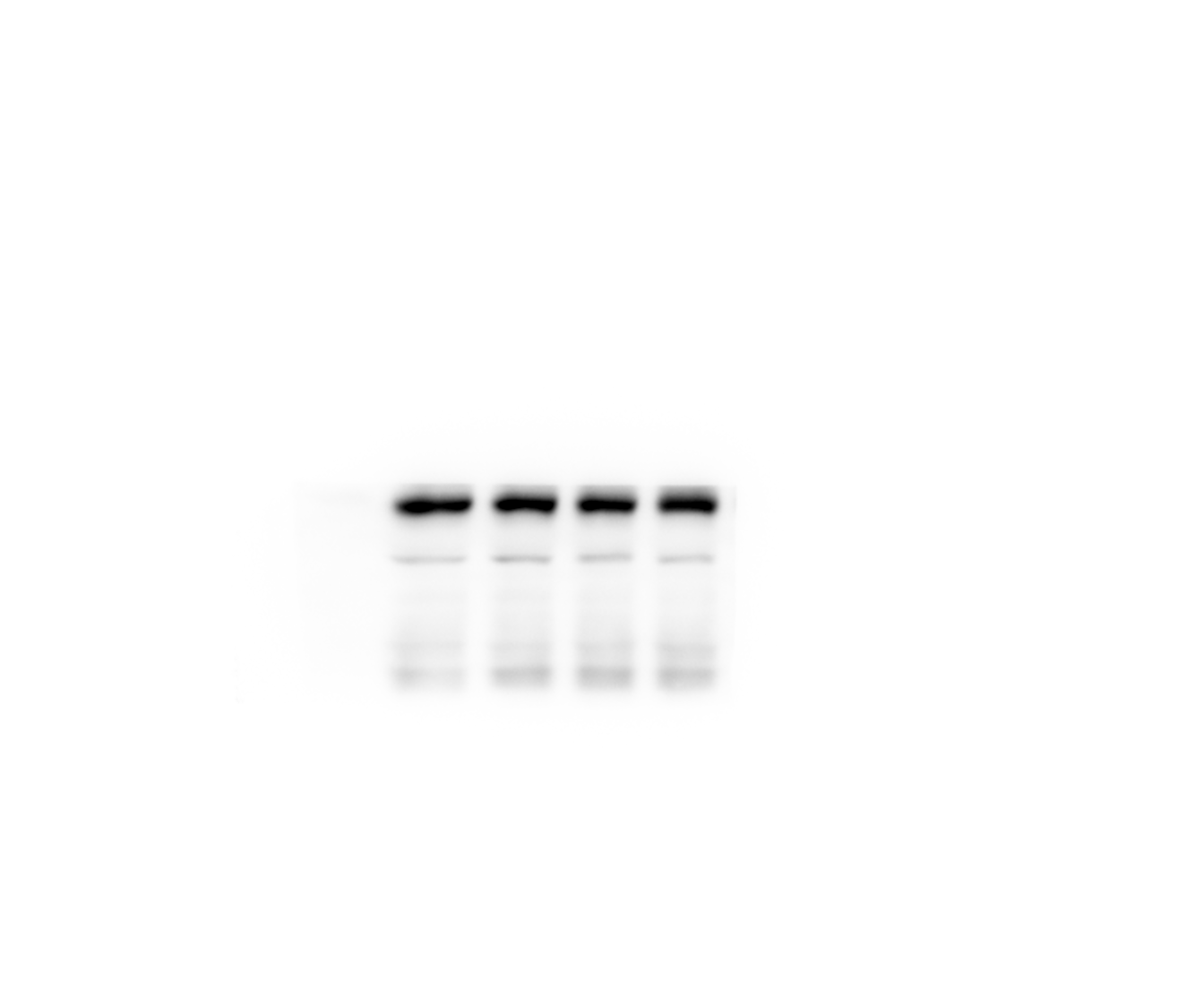


**Supplementary Figure 6.** WB raw data of p38


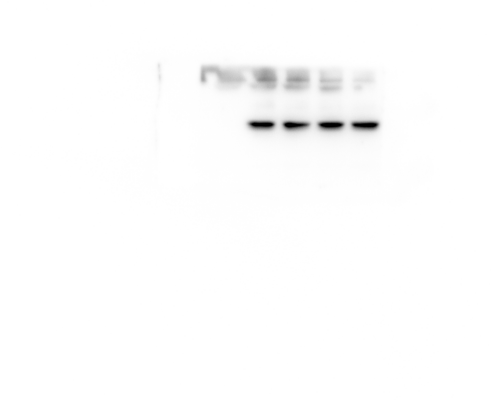


**Supplementary Figure 7.** WB raw data of Tublin
